# Association between late bedtime and obesity in children and adolescents: a meta-analysis

**DOI:** 10.3389/fped.2024.1342514

**Published:** 2024-03-15

**Authors:** Na Hu, Ying Wu, Qing Yao, Shixiang Huang, Wen Li, Zhenhua Yao, Chunfeng Ye

**Affiliations:** Department of Pediatrics, The Second Affiliated Hospital of Nanchang University, Nanchang, China

**Keywords:** sleep, bedtime, obesity, children, adolescents, meta-analysis

## Abstract

**Background:**

Short sleep duration has been related to obesity in children and adolescents. However, it remains unknown whether late bedtime is also associated with obesity and whether the association is independent of sleep duration. A meta-analysis was performed to address this issue.

**Methods:**

In order to accomplish the aim of the meta-analysis, a comprehensive search was conducted on databases including PubMed, Embase, and Web of Science to identify observational studies. The cutoff to determine late bedtime in children in this meta-analysis was consistent with the value used among the included original studies. As for obesity, it was typically defined as a body mass index (BMI) > 95th percentile of age and sex specified reference standards or the International Obesity Task Force defined age- and gender-specific cut-off of BMI. The Cochrane *Q* test was employed to evaluate heterogeneity among the included studies, while the *I*^2^ statistic was estimated. Random-effects models were utilized to merge the results, considering the potential impact of heterogeneity.

**Results:**

Tweleve observational studies with 57,728 participants were included. Among them, 6,815 (11.8%) were obese. Pooled results showed that late bedtime reported by the participants or their caregivers was associated with obesity (odds ratio [OR]: 1.27, 95% confidence interval [CI]: 1.16–1.39, *p* < 0.001; *I*^2^ = 0%). Subgroup analysis showed consistent results in studies with (OR: 1.33, 95% CI: 1.04–1.70, *p* = 0.02) and without adjustment of sleep duration (OR: 1.27, 95% CI: 1.14–1.41, *p* < 0.001). Further subgroup analysis also showed that the association was not significantly affected by study location, design, age of the participants, or diagnostic methods for obesity (*p* for subgroup difference all >0.05).

**Conclusion:**

Late bedtime is associated with obesity in children and adolescents, which may be independent of sleep duration.

## Introduction

Childhood obesity has emerged as a significant global public health concern, attributable to the evolving lifestyles of individuals in both developed and developing nations (1, 2). The escalating prevalence of childhood obesity is indicative of an ongoing trend (3, 4). The findings of the 2019/2020 English National Child Measurement Program indicate that 9.9% of children aged 4–5 were classified as obese (5). Upon conducting a subsequent assessment of the same cohort at age 10–11, it was observed that the prevalence of obesity had escalated to 21% (5). Similarly, a recent systematic review conducted in China revealed a persistent and heterogeneous prevalence of overweight and obesity among children and adolescents in the country (6). The presence of obesity in children and adolescents has been linked to heightened lifetime susceptibility to metabolic disorders (7, 8), cardiovascular diseases (9, 10), and cancer (7, 11). Consequently, it is imperative to identify the risk factors associated with childhood obesity, particularly those related to unhealthy lifestyles, in order to improve the overall body weight status within this population (12, 13).

Accumulating evidence indicates that children and adolescents with insufficient nighttime sleep are at a heightened risk of childhood obesity, in contrast to those with sufficient sleep duration (14, 15). In recent decades, insufficient nighttime sleep has been closely related to delayed bedtime in both adults and children, which are likely to be caused by various external factors, such as social interactions, food, entertaining activity, and increased screen time etc. Nevertheless, prior investigations examining the correlation between delayed bedtime and obesity in children have yielded inconclusive findings (16). Furthermore, given the close interrelation between bedtime and sleep duration, it remains uncertain whether the potential association between delayed bedtime and obesity is independent of the impact of inadequate sleep duration (17). In light of this dearth of knowledge, a meta-analysis of observational studies was conducted to evaluate the potential association between late bedtime and obesity in children and adolescents.

## Materials and methods

The study adhered to the Meta-analyses Of Observational Studies in Epidemiology (MOOSE) guideline (18) and the Cochrane Handbook (19) throughout the stages of planning, conducting, and reporting.

### Inclusion and exclusion criteria of studies

The development of inclusion criteria adhered to the Participant, Intervention, Comparison, Outcome, and Study design (PICOS) recommendations and aligned with the objective of the meta-analysis.

P (participants): Children and adolescents from general population or community population.

I (exposure): Late bedtime. To the best of our knowledge, a universal cutoff has not been developed and it is likely to be varied according to the age of the children. Because we are focusing on the comparison between children with early vs. late bedtime, the cutoff to determine late bedtime in children in this meta-analysis was consistent with the value used among the included original studies.

C (control): Early bedtime. The cutoff to define children and adolescents with early bedtime were consistent with that used among the included studies.

O (outcomes): The prevalence or the incidence of obesity compared between children and adolescents with later vs. early bedtime. The diagnostic methods and criteria for obesity were in accordance with that used in the original studies. As for obesity, it was typically defined as BMI > 95th percentile of age and sex specified reference standards or the International Obesity Task Force (IOTF) defined age- and gender-specific cut-off of BMI. For studies reporting multiple categories of participants with different late bedtime, those with the latest bedtime were included for analysis.

S (study design): This study incorporated observational studies, such as case-control study, cross-sectional study, or cohort study. Excluded from the meta-analysis were reviews, editorials, meta-analyses, and studies that included children or adolescents with specific clinical conditions, studies that included adult population, did not evaluate the influence of bedtime, or did not report the outcome of obesity. In cases where there was an overlap in populations, the study with the largest sample size was included in the meta-analysis.

### Search of databases

A comprehensive search was conducted in electronic databases, namely PubMed, Embase, and Web of Science, encompassing the period from inception to August 10, 2023. The search strategy employed relevant terms pertaining to the subject matter of our investigation, aiming to identify studies published within this timeframe, which included: (1) “sleep time” OR “bed time” OR “bedtime” OR “late sleep” OR “late-time sleeping” OR “late time sleeping” OR “sleep timing”; (2) “obesity” OR “obese”; and (3) “child” OR “children” OR “adolescents” OR “pediatric” OR “pediatric” OR “preschool” OR “toddler”. Only studies that met the criteria of being published as full-length articles in English or Chinese and appearing in peer-reviewed journals were included in our analysis. Additionally, during our manual screening process, we thoroughly examined the references cited in relevant original and review articles to identify any potentially relevant studies.

### Data extraction and quality evaluation

Two authors independently performed literature searches, data collection, and assessments of study quality. In cases where discrepancies emerged, discussions between the two authors were indicated to come to a consensus. The analysis of studies encompassed the gathering of data related to study information, design characteristics, sample size, demographic factors of the participants, methods and cutoffs for evaluating bedtime, follow-up durations for cohort studies, diagnostic methods for obesity, number of participants with obesity, and potential confounding variables adjusted when the association between late bedtime and obesity were analyzed. The quality of the study was assessed using the Newcastle-Ottawa Scale (NOS) (20). For cohort studies, this scale judges the quality regarding three aspects: selection of the study groups; the comparability of the groups; and the ascertainment of the outcome of interest. For case-control and cross-sectional studies, this scale assess the quality of the studies also via three aspects: selection and definitions of cases and controls, comparability between cases and controls, and ascertainment of expose between cases and controls. The NOS varied between one to nine stars, with a higher star indicating a better study quality.

### Statistics

Odds ratios (ORs) and their corresponding 95% confidence intervals (CIs) were utilized as the variables to assess the relationship between late bedtime and obesity in children and adolescent. To stabilize and normalize the variance, a logarithmical transformation was applied to the OR and its corresponding standard error in each study (21). The Cochrane *Q* test and the *I*^2^ statistic (22) were employed to estimate between-study heterogeneity. A value of *I*^2^ greater than 50% indicates the presence of significant heterogeneity among the studies. The random-effects model was utilized to combine the findings, as it has been recognized to account for potential heterogeneity (19). Sensitivity analysis excluding one dataset at a time was performed to evaluate the robustness of the finding. Additionally, predefined subgroup analysis was conducted to explore whether the results were significantly affected by characteristics such as study country, design, age of the participants, definition of obesity, or adjustment of sleep duration. Publication bias was estimated using a funnel plot, which involved visual assessments of symmetry, as well as Egger's regression asymmetry test (23). A *p* value < 0.05 was considered as statistically significant. This applies to the statistical in meta-analysis and the result of the Egger's regression test The statistical analyses were conducted using RevMan (Version 5.1; Cochrane Collaboration, Oxford, UK) and Stata software (version 12.0; Stata Corporation, College Station, TX).

## Results

### Database search and study retrieval

**Figure 1:**
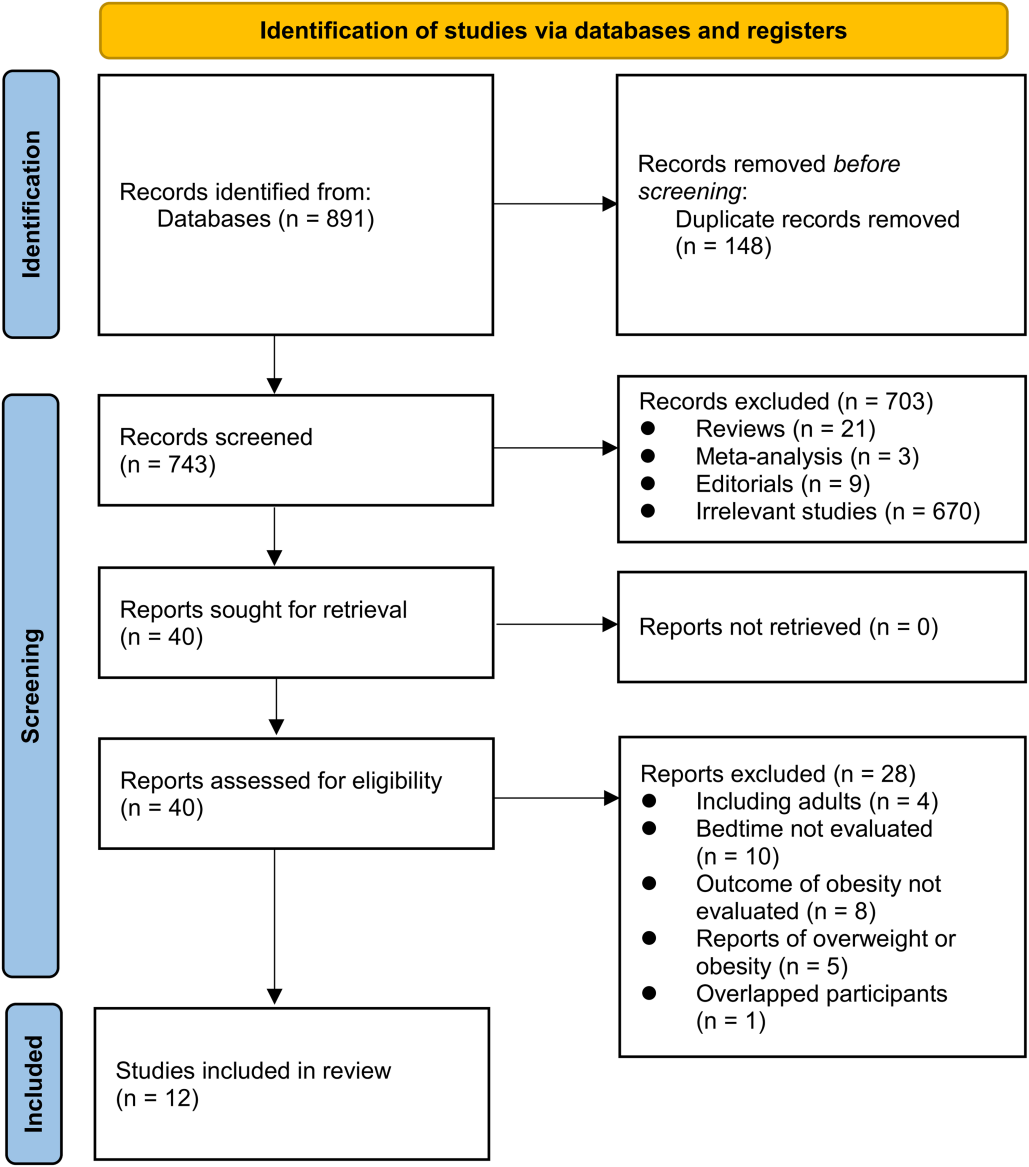
Flowchart of database search and study inclusion.

Figure 1 illustrates the procedure employed for conducting the literature search and study retrieval. Initially, a total of 891 records were acquired from the designated database, and subsequently, 148 duplicate entries were eliminated. Upon scrutinizing the titles and abstracts, an additional 703 studies were excluded due to their incompatibility with the objectives of the meta-analysis. Following comprehensive evaluations of the full texts of 40 studies, 28 were excluded based on the rationales outlined in Figure 1. Consequently, twelve studies were deemed suitable for the subsequent meta-analysis (24–35).

### Study characteristics

The characteristic of the studies are summarized in Table 1. Overall, 12 observational studies, including eight cross-sectionals studies (24–27, 29, 30, 33, 35) and four cohort studies (28, 31, 32, 34) were included. These studies were published between 2009 and 2023, and performed in China, Australia, Canada, the United States, Italy, and New Zealand. A total of 57,728 children and adolescents were included. The bedtime of the participants were self-reported or reported by their caregivers. As for the cutoffs for refining late bedtime, medians of sleep time of the included participants were used in three studies (25, 32, 33), while in the other eight studies, cutoffs of later than 9:00 pm (28, 34), 9:30 pm (27), 10:00 pm (24, 26, 30, 35), 11:00 pm (29), or midnight (31) were selected. The diagnosis of obesity in children and adolescents was made if the body mass index (BMI) > 95th percentile according to age and gender using the local children growth charts or using the IOTF defined age- and gender-specific cut-off of BMI. Accordingly, 6,815 (11.8%) of the participants were obese. Univariate analyses were performed in two studies (24, 29), while for the other ten studies, age, sex and other confounding factors were incorporated in the multivariate analysis (25–28, 30–35). The NOS of the included studies were seven to nine, indicating that they were of good quality (Table 2).

**Table 1:** Characteristics of the included studies.

Study	Location	Design	No. of participants	Age range (years)	Male (%)	Methods for evaluation of bedtime	Cutoff of late bedtime	Follow-up duration (years)	Definition of OB	Number of participants with OB	Variables adjusted
Jiang et al. (24)	China	CS	1,311	3∼4	50.3	Caregiver reported	>10 pm	NA	OB: BMI > 95th percentile	113	None
Olds et al. (25)	Australia	CS	2,200	9∼16	49.4	Self-reported	>Media*n* (NR)	NA	OB: IOTF defined age- and gender-specific cut-off of BMI	143	Age, sex, household income, remoteness, and sleep duration
Khan et al. (26)	Canada	CS	5,560	10∼11	48	Caregiver reported	>10 pm	NA	OB: IOTF defined age- and gender-specific cut-off of BMI	667	Age, sex, sleep duration, snoring, household income, parental education attainment, and place of residence.
Scharf and DeBoer (27)	USA	CS	15,950	4∼5	50.8	Caregiver reported	>9:30 pm	NA	OB: BMI > 95th percentile	2,460	Age, sex, race/ethnicity, SES, and television viewing
Anderson et al. (28)	USA	PC	977	3∼5	50	Mother reported	>9 pm	10	OB: BMI > 95th percentile	161	Age, sex, race/ethnicity, birth weight, maternal education, maternal sensitivity at preschool age, and maternal obesity
Ferranti et al. (29)	Italy	CS	1,586	11∼14	54.9	Self-reported	>11 pm	NA	OB: IOTF defined age- and gender-specific cut-off of BMI	389	None
Jiang et al. (30)	China	CS	14,946	3∼6	53.7	Caregiver reported	>10 pm	NA	OB: BMI > 95th percentile	1,562	Age, sex, residence, parental BMI, parental education, dietary category, outdoor activity, screen time, and sleep duration
Lim et al. (31)	China	PC	516	6∼18	37.6	Caregiver reported or self-reported	>0 am	6.2	OB: BMI > 95th percentile	58	Age and sex
Venkatapoorna et al. (33)	New Zealand	CS	169	6∼10	49.1	Caregiver reported	>Median (8:30 pm)	NA	OB: IOTF defined age- and gender-specific cut-off of BMI	28	Age, sex, television exposure, and dinnertime
Roy et al. (32)	New Zealand	RC	1,642	2∼5	51.2	Caregiver reported	Median (2, 3, and 5 year groups > 8:00, 7:30, and 7:47 respectively)	5	OB: BMI > 95th percentile	62	Age, sex, maternal tertiary education, maternal age at birth, sleep intervention, and study
Reyna-Vargas et al. (34)	Canada	PC	2,185	3	53	Caregiver reported	>9 pm	2	OB: BMI > 95th percentile	125	Age, sex, birth weight, breastfeeding status, maternal education, daily caloric intake, maternal perceived stress score, 5 years’ physical activity, and maternal BMI
Yang et al. (35)	China	CS	10,686	9∼18	50.5	Self-reported	>10 pm	NA	OB: IOTF defined age- and gender-specific cut-off of BMI	1,047	Age, sex, parental educational levels and physical activity levels

OB, obesity; CS, cross-sectional; PC, prospective cohort; RC, retrospective cohort; NA, not applicable; BMI, body mass index; IOTF, International Obesity Task Force; SES, socioeconomical status; NR, not reported.

**Table 2:** Quality evaluation of the included observational studies via the Newcastle-Ottawa scale.

Cross-sectional study	Adequate definition of cases	Representativeness of cases	Selection of controls	Definition of controls	Control for age and sex	Control for other confounders	Exposure ascertainment	Same methods for events ascertainment	Non-response rates	Total
Jiang et al. (24)	1	1	1	1	0	0	1	1	1	7
Olds et al. (25)	1	1	1	1	1	1	1	1	0	8
Khan et al. (26)	1	1	1	1	1	1	1	1	1	9
Scharf and DeBoer (27)	1	1	1	1	1	1	1	1	1	9
Ferranti et al. (29)	1	1	1	1	0	0	1	1	1	7
Jiang et al. (30)	1	1	1	1	1	1	1	1	1	9
Venkatapoorna et al. (33)	1	1	1	1	1	1	1	1	0	8
Yang et al. (35)	1	1	1	1	1	1	1	1	1	9
Cohort study	Representativeness of the exposed cohort	Selection of the non-exposed cohort	Ascertainment of exposure	Outcome not present at baseline	Control for age and sex	Control for other confounding factors	Assessment of outcome	Enough long follow-up duration	Adequacy of follow-up of cohorts	Total
Anderson et al. (28)	0	1	1	1	1	1	1	1	1	8
Lim et al. (31)	1	1	1	1	1	0	1	1	1	8
Roy et al. (32)	0	1	1	1	1	0	1	1	1	8
Reyna-Vargas et al. (34)	1	1	1	1	1	0	1	1	1	9

## Results of meta-analysis

**Figure 2:**
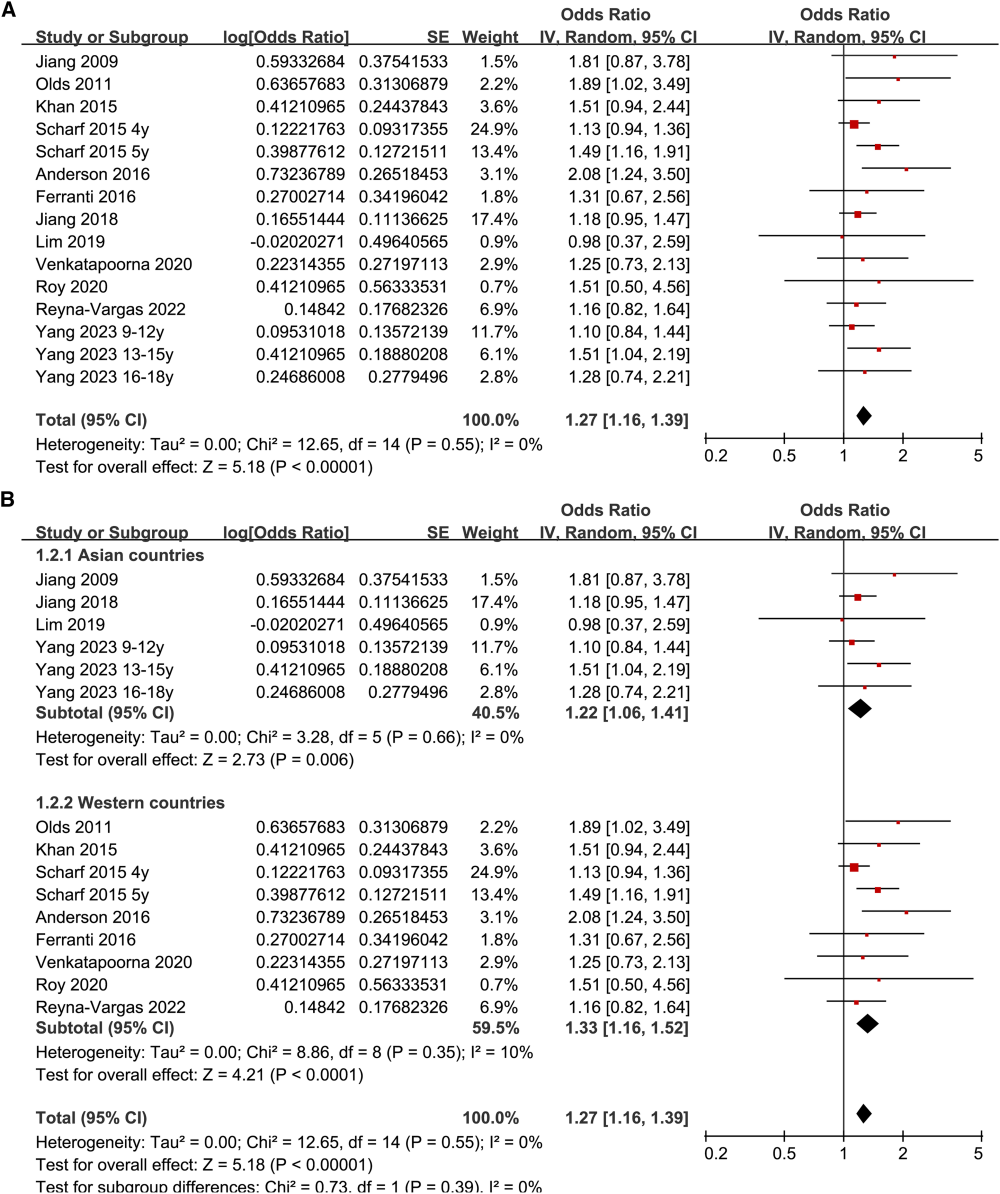
Forest plots for the meta-analyses regarding the association between late bedtime and obesity in children and adolescents; (**A**) overall meta-analysis; and (**B**) subgroup analysis according to study country.

**Figure 3:**
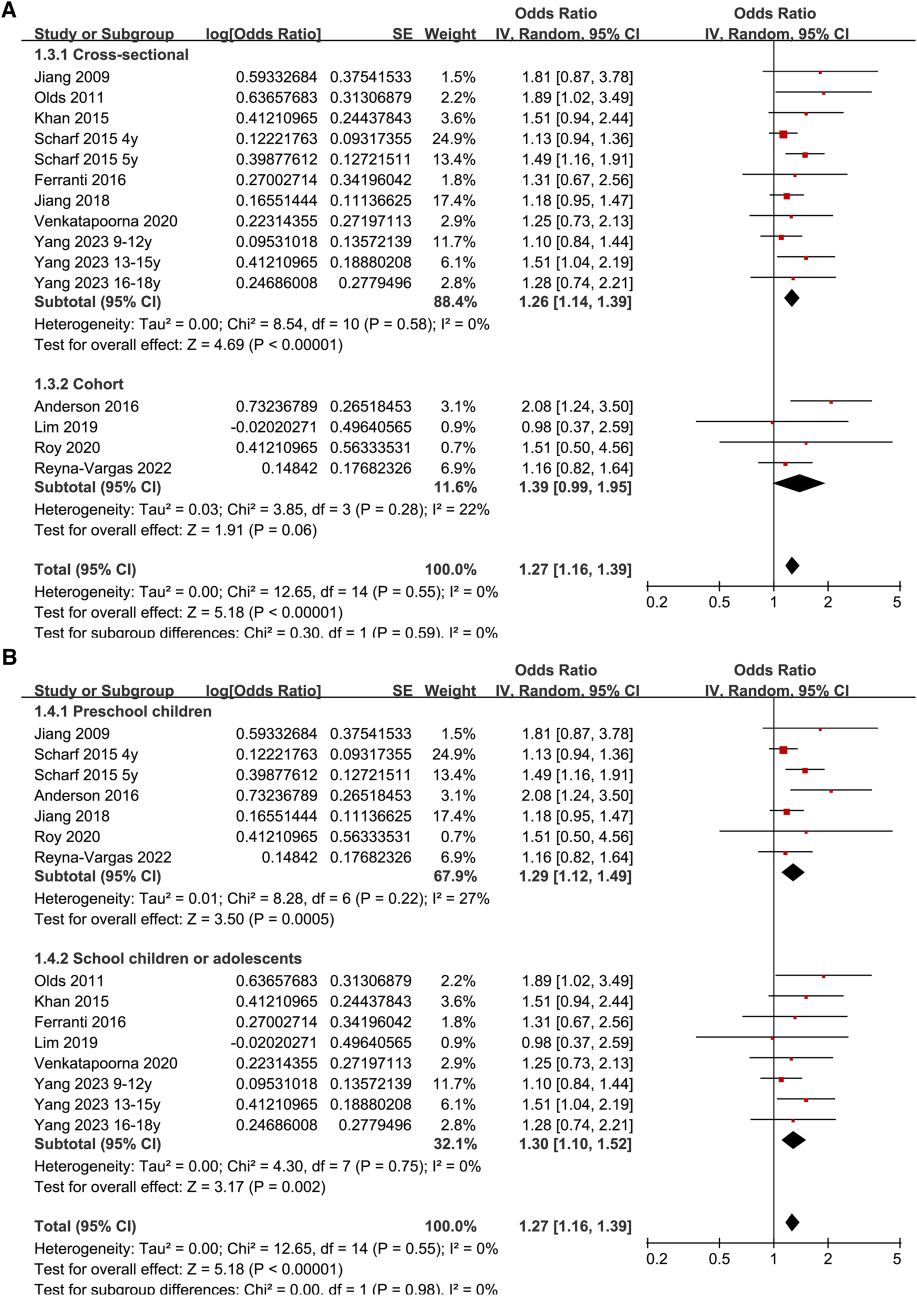
Forest plots for the subgroup-analyses regarding the association between late bedtime and obesity in children and adolescents; (**A**) subgroup analysis according to study design; and (**B**) subgroup analysis according to age group.

**Figure 4:**
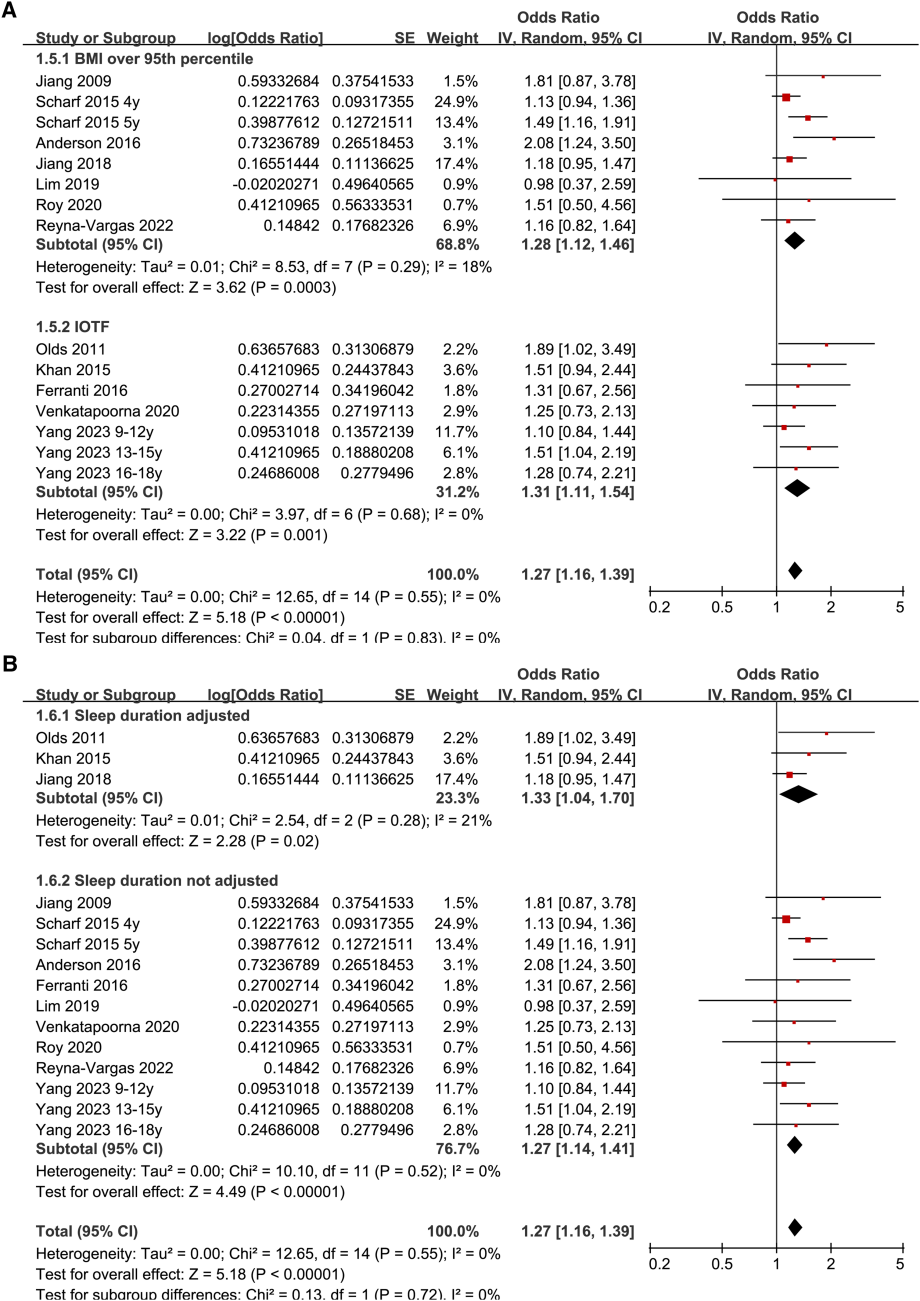
Forest plots for the subgroup-analyses regarding the association between late bedtime and obesity in children and adolescents; (**A**) subgroup analysis according to diagnostic methods for obesity; and (**B**) subgroup analysis according to the adjustment of sleep duration.

Since two studies reported the results according to the age group of the included children and adolescents (27, 35), these datasets were included in the meta-analysis independently. Overall, 15 datasets from 12 observational studies were involved in the meta-analysis. Pooled results showed that late bedtime was significantly associated with obesity in children and adolescents (OR: 1.27, 95% CI: 1.16–1.39, *p* < 0.001; Figure 2A) with no significant heterogeneity (*I*^2^ = 0%). Sensitivity analyses by omitting one dataset at a time showed consistent results (OR: 1.24–1.32, *p* all < 0.05). In addition, sensitivity analysis limited to studies with multivariate analyses (25–28, 30–35) showed similar results (OR: 1.26, 95% CI: 1.15–1.39, *p* < 0.001; *I*^2^ = 0%). Subgroup analysis showed consistent results in studies of Asian and Western countries (*p* for subgroup difference = 0.39, Figure 2B), in cross-sectional and cohort studies (*p* for subgroup difference = 0.59, Figure 3A), in preschool children and school children and adolescents (*p* for subgroup difference = 0.98, Figure 3B), and in studies of obesity defined as BMI > 95th percentile or according to the IOTF criteria (*p* for subgroup difference = 0.83, Figure 4A). Specifically, subgroup analysis showed consistent results in studies with (OR: 1.33, 95% CI: 1.04–1.70, *p* = 0.02) and without the adjustment of sleep duration (OR: 1.27, 95% CI: 1.14–1.41, *p* < 0.001; *p* for subgroup difference = 0.72; Figure 4B).

### Publication bias

**Figure 5:**
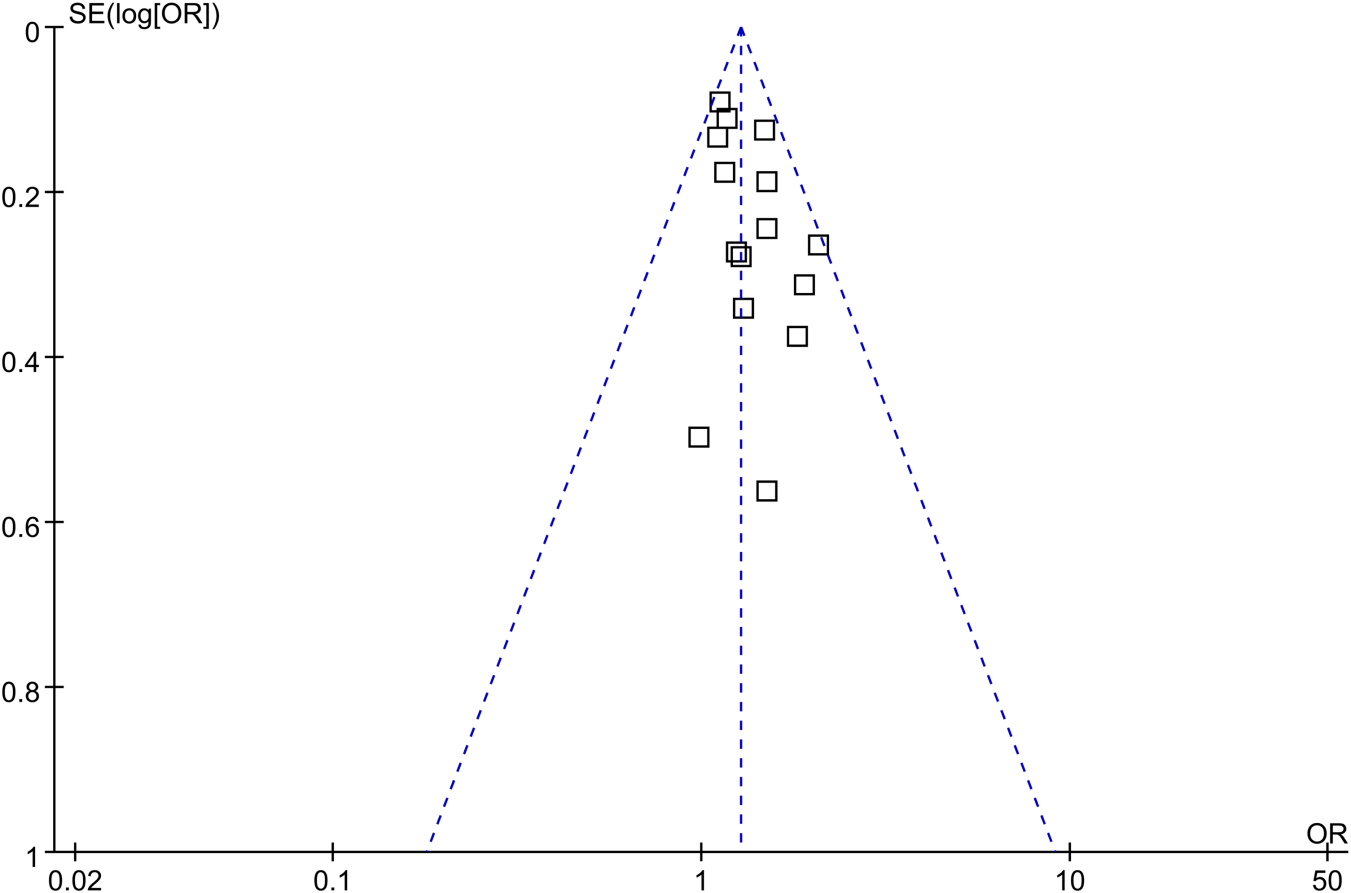
Funnel plots for the publication bias underlying the meta-analysis of the association between late bedtime and obesity in children and adolescents.

The funnel plots depicting the meta-analyses of the association between late bedtime and obesity in children and adolescents are presented in Figure 5. Upon visual inspection, the plots exhibit symmetrical patterns, indicating a minimal presence of publication bias. Furthermore, the application of Egger's regression test yielded *p*-value of 0.75, further supporting the notion of a low probability of publication bias.

## Discussion

In this meta-analysis, we combined the results of 15 datasets from 12 eligible observational studies and the results showed that compared to participants with early bedtime, children and adolescents with late bedtime were significantly associated with the prevalence and incidence of obesity. Further sensitivity analyses by excluding one dataset at a time showed consistent results. In addition, subgroup analyses showed that the association between late bedtime and obesity in children and adolescents was not significantly affected by study country, study design, age group of the participants, and diagnostic methods for obesity. Particularly, the association was consistent in studies with and without the adjustment of sleep duration. Taken together, results of the meta-analysis indicate that late bedtime is associated with obesity in children and adolescents, which may be independent of the influence of sleep duration.

As far as we know, this study may be the first meta-analysis regarding the association between bedtime and obesity in children and adolescents. There are some methodological advantages of the meta-analysis. We performed extensive literature search in three commonly used literature databases and retrieved the up-to-date literatures that evaluated the association between bedtime and obesity. Ten of the included studies were based on data of multivariate analyses, and a sensitivity analysis limited to these studies showed similar results, which suggested a potentially independent association between late bedtime and obesity in children and adolescents. Subsequent sensitivity analyses by excluding one dataset at a time suggested that the results were not primarily driven by either of the included studies, reflecting the stability of the results. Finally, subgroup analysis showed that the association between late bedtime and obesity was not likely to be significantly affected by study characteristics such as study country, design, age group, definition of obesity, or adjustment of sleep duration. The latter is particularly important because inadequate sleep duration has been established as a risk factor of obesity in children (36), and late bedtime is closely related to short sleep duration. These findings support that besides short sleep duration, late bedtime may also be a risk factor of obesity in children and adolescents. These findings are important to designate optimal lifestyle modification regarding sleep habit to reduce the risk of obesity in children.

The association between late bedtime and obesity in children and adolescents may be influenced by various mechanisms. One potential factor is the alteration of dietary habits in this population. A study conducted on 93 adolescents in the United States demonstrated that late bedtime and sleep deprivation were linked to increased consumption of carbohydrates, added sugars, foods with higher glycemic load, and sweet beverages. Conversely, there was a decrease in the intake of fruits and vegetables, which could potentially contribute to adverse weight and cardiometabolic outcomes (37). A subsequent randomized cross-over study indicated that children who had a later bedtime consumed an additional 35 calories per day from sugar-sweetened beverages during periods of decreased sleep, compared to controls that had an earlier bedtime (38). These instances of late bedtime were more likely to occur after 8 pm (38). In addition to its impact on dietary patterns, late bedtime has also been linked to prolonged screen time before sleep (39), decreased physical activity (40), and the development of insulin resistance (41). These factors may also contribute to the connection between late bedtime and obesity. Besides, increased screen time and television time before bed has also been associated with late bedtime in children (42, 43). In addition, these two factors, together with room light before beds have all been related to the suppressed melatonin onset and shortened melatonin duration (44). Interestingly, endogenous melatonin has been shown to protect against body weight gain induced by sleep deprivation in a previous study (45), suggesting change in endogenous melatonin may be a key mechanisms underlying the association between late bedtime and obesity in children. However, the specific mechanisms and molecular pathways that underlie this association remain to be clarified in future studies.

This study has several limitations that should be acknowledged. Firstly, the protocol of the meta-analysis was not prospectively registered. Secondly, the majority of the studies included in this analysis were cross-sectional and retrospective, which introduces the possibility of selection and recall biases. To validate the findings, it is necessary to conduct large-scale prospective studies. Thirdly, the optimal cutoff of early bedtime regarding the influence of bedtime on obesity should be investigated in future studies. Moreover, in view of the close relationship between bedtime and sleep duration, these factors should be both considered in future studies. Furthermore, although most of the included studies employed multivariate regression analyses to determine the association, it is important to acknowledge that the influence of residual factors on the correlation between late bedtime and obesity in this population. Besides, the differences in cutoff of late bedtime and adjusted variables among the included studies may affect the results of the meta-analysis and lead to potential heterogeneity. Ultimately, due to the nature of this meta-analysis being based on observational studies, it is not possible to establish a causal relationship between late bedtime and obesity in children and adolescents.

## Conclusions

The findings of the meta-analysis indicate that there is a significant correlation between late bedtime and obesity in children and adolescents, potentially unaffected by the duration of sleep. These results propose late bedtime as a plausible risk factor for obesity within this specific demographic. Consequently, future research should consider both bedtime and sleep duration as crucial factors when determining the most effective lifestyle modifications for reducing the risk of obesity in children and adolescents.

## Data Availability

The original contributions presented in the study are included in the article/Supplementary Material, further inquiries can be directed to the corresponding author.
